# The Photobiology of Microbial Pathogenesis

**DOI:** 10.1371/journal.ppat.1000470

**Published:** 2009-11-26

**Authors:** Alexander Idnurm, Sean Crosson

**Affiliations:** 1 Division of Cell Biology and Biophysics, School of Biological Sciences, University of Missouri-Kansas City, Kansas City, Missouri, United States of America; 2 Department of Biochemistry and Molecular Biology, and The Committee on Microbiology, The University of Chicago, Chicago, Illinois, United States of America; University of California San Francisco, United States of America

## Why Is Light Important for Pathogenic Microbes?

**Figure 1:**
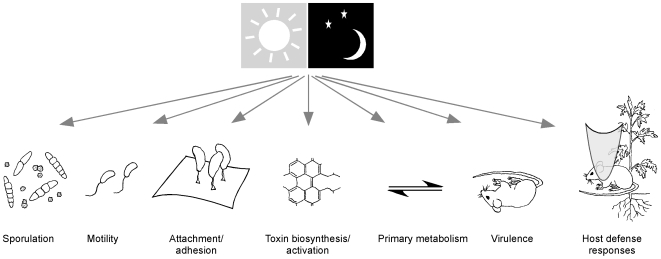
Effects of light on microbial pathogenesis. Light (or its absence) represents an environmental signal that is known to regulate many properties of a microbial cell, which may indirectly or directly influence the development of disease.

Light is an abundant signal that many organisms use to assess the status of their environment. Species from all kingdoms have evolved the capacity to sense and respond to wavelengths across the visible spectrum. Light has long been linked to disease (Figure 1); however, the mechanisms behind many of these observations are not well understood. Recently, a direct link has been established between specific protein photosensors and the ability to cause disease in both pathogenic bacteria and fungi [1]–[3]; thus, certain pathogens require these photosensors for full virulence. A role for photoperception is likely to emerge as a common theme in microbial pathogenesis.

## What Photosensors Are Known in Microbes That Could Regulate Virulence?

**Figure 2:**
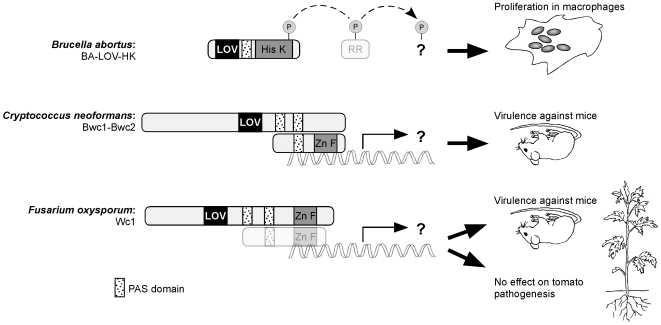
LOV domain photosensors are required for pathogenesis. Structure of the photosensors from *Brucella abortus*, *Cryptococcus neoformans*, and *Fusarium oxysporum*. The signal transduction pathway is unknown in all three cases, but is likely to involve a phosphorylation cascade including downstream signaling partners in *B. abortus* and a transcriptional response for the two fungal species. In the case of *B. abortus*, mutation of the photoreceptor gene affects proliferation in macrophages, while photoreceptor mutation in the two pathogenic fungi species modulates the rate at which mice succumb to infection. A *wc-2* homlog of *F. oxysporum* is predicted (FOXG_01037.2) but has yet to be characterized from this species.

Microbes have sensory proteins that can perceive a range of energies across the visible spectrum, as well as the far ultraviolet (UV) and infrared wavelengths [4]. These protein photosensors fall into distinct classes depending on the chromophore that they bind, i.e., the cofactor that actually does the light sensing. Microbial photosensors include the phytochromes, cryptochromes, rhodopsins, photoactive yellow protein, and flavoproteins with BLUF (blue light sensing using FAD; [5]) or LOV (light, oxygen or voltage; [6]) domains. At present it is the blue light photoreceptors that are known to be required for virulence in microbes. In these cases, the photosensor proteins all contain a LOV domain that in the dark non-covalently binds a flavin cofactor (Figure 2). Light absorption by the flavin initiates formation of a covalent bond between a conserved cysteine residue in the LOV domain and the 4a carbon of the flavin [7]. Formation of this bond alters the conformation of the LOV domain, resulting in signal transmission. The output affected by the LOV domain can vary: in the bacterial systems discussed below, absorption of blue light via the LOV domain modulates the activity of a histidine kinase domain [2],[8], whereas in the fungi the LOV domain is predicted to affect gene expression via allosteric regulation of a zinc finger domain that binds DNA [9] (Figure 2). While it is only the LOV domain proteins that are currently established as regulators of microbial virulence, it will be of interest to test other photosensors for their role in pathogenesis.

## What Do We Currently Know about Photoregulation of Bacterial Pathogenesis?

Probably the most surprising example of photoregulation of bacterial pathogenesis was discovered by Swartz and colleagues, who demonstrated that proliferation of the Gram-negative pathogen *Brucella abortus* in a macrophage infection model requires exposure to visible light [2]. Moreover, this research team showed that the light dependence of cellular proliferation in macrophages required a blue light photosensory histidine kinase, which they named LOV-HK (Figure 2). Why virulence is regulated by visible light in this pathogen remains unresolved, as is the molecular/cellular mechanism underlying the light-dependent regulation of virulence. However, work on a photosensory LOV histidine kinase in the related species *Caulobacter crescentus* may provide some insight into light-regulated virulence in *B. abortus*. Specifically, activation of the photosensory two-component system LovK-LovR in *C. crescentus* results in modulation of the adhesive capacity of the cell [8]. Certainly, the ability of bacterial pathogens to adhere to host cells is often a critical determinant of virulence, and it will be interesting to see if the adhesive response observed in *C. crescentus* is conserved in its relative *B. abortus*, or in other pathogens that encode LOV histidine kinases (e.g., the plant pathogen *Pseudomonas syringae*). Bioinformatic analysis of over 600 bacterial genomes reveals that at least one LOV domain protein is present in 13% of species, including a number of pathogens [10]. The observations from *B. abortus* may therefore be widely relevant. Finally, it was recently reported that white light has a repressive effect on both expression of the flagellar genes *flaA*, *flaB*, and *flaC* and on cell adhesion and virulence in the plant pathogen *Agrobacterium tumefaciens* [11]. This photoresponse was independent of the three photoreceptors that can be identified in the genome sequence of *A. tumefaciens*, including two phytochromes and one photolyase/cryptochrome. An exciting possibility is that novel visible light photoreceptors are regulating these processes.

## Does Light Also Influence Fungal Pathogenesis?

Sequencing projects reveal that fungal genomes encode putative photosensory proteins of the rhodopsin, phytochrome, cryptochrome, and LOV domain classes. A fungal LOV domain photosensor, first identified and named WHITE COLLAR 1 in the non-pathogen *Neurospora crassa*, is present throughout most of the kingdom, suggesting an ancient origin. In all species examined, WC-1 physically interacts with a second protein, WC-2, that contains a zinc finger DNA-binding domain such that the complex can act as a light-sensitive transcription factor (Figure 2). One fungus in which a *wc-1* homolog is required for virulence is *Cryptococcus neoformans*, a cause of fatal meningitis in humans. Light regulates the filamentous mating process of the fungus via the WHITE COLLAR homologs Bwc1 and Bwc2. Mutation of these genes causes a reduction in resistance to UV light, and also renders the strains less virulent in a mouse inhalation model of the disease [1],[12]. *Fusarium oxysporum* is a filamentous species best known as a plant pathogen but is also a cause of human disease: its *wc-1* homolog in a tomato isolate is required for full virulence in a mouse tail-vein injection model, but surprisingly has no effect on virulence in tomato roots [3]. Finally, there is tantalizing evidence suggesting that exposure to light influences virulence in several insect pathogens and the human pathogen *Histoplasma capsulatum* [13]. The cellular/molecular mechanism of light regulation of virulence in all fungal species is unknown.

## How Can I Shed Light on the Photobiology of My Favorite Pathogen?

Clearly, changes in light quantity (i.e., fluence) or quality (i.e., wavelength) represent a cue in the regulation of virulence in select microbial pathogens. As described above, there are only three reports of photoreceptors that specifically affect virulence and the underlying mechanisms are unknown; thus, the field is wide open for new investigations. Grow your favorite microbe in the light and dark and see if it does anything differently. Or search the genome sequence for candidate photosensors, mutate these genes, and test the ability of the mutant strains to cause disease. There are several caveats to consider in microbial photobiology experiments: 1) it is likely that certain photosensory genes or pathways may have accumulated mutations over years of laboratory cultivation and are no longer functional, 2) the chemical properties of growth media can change on being exposed to bright visible light, and 3) proteins other than bona fide photosensory receptors also bind cofactors that absorb in the visible region of the spectrum and their function may be affected by light [14]. Other things one should consider when measuring the effects of light on virulence include logistical difficulties in controlling the light environment in certain plant and animal facilities, and the possibility that light also affects host defenses against microbes [15],[16]. Despite these experimental challenges, the continued impact of diseases worldwide, the rise in antibiotic resistance, and the emergence of new pathogens underscores the need for better understanding the basis of microbial pathogenesis. It is time to put microbial light sensing under the spotlight.
